# Supplementary material for: Human Fibroblast Growth Factor-Treated Adipose-Derived Stem Cells Facilitate Wound Healing and Revascularization in Rats with Streptozotocin-Induced Diabetes Mellitus
Source: Cells. 2023 Apr 13;12(8):1146. doi: 10.3390/cells12081146 (PMC10136967; doi:10.3390/cells12081146)
Supplement: Supplementary file 1 [file cells-12-01146-s001.zip › cells-2186011-supplementary.pdf]

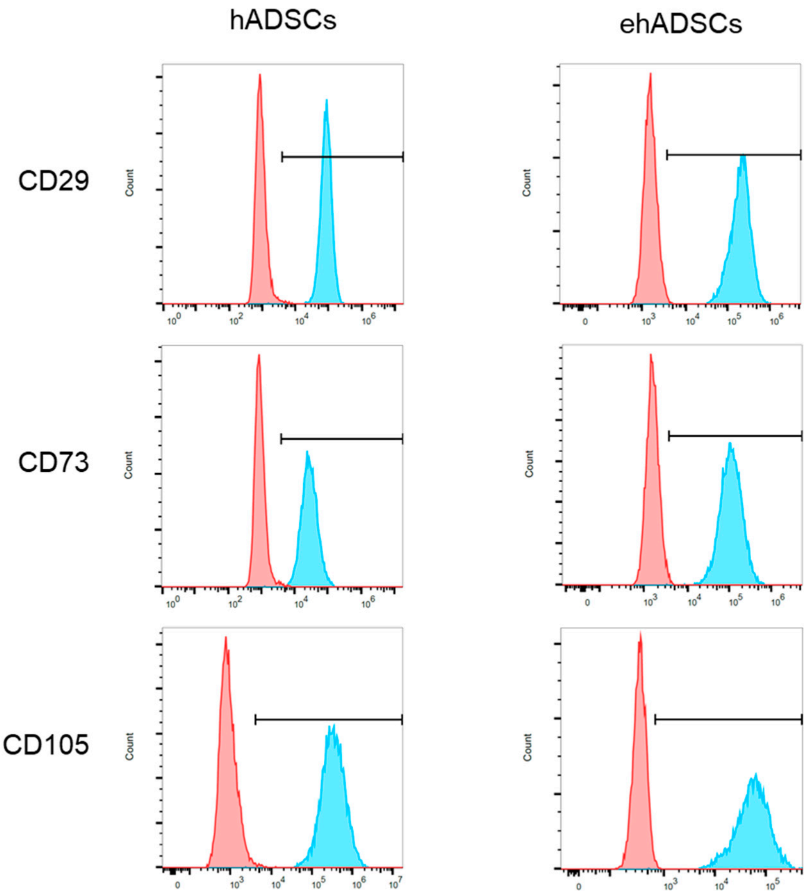

Supplemental Figure S1. Characterization of hADSCs and ehADSCs using flow cytometry on ADSC markers, such as CD29, CD73, and CD105. Red histograms indicate cell stained with isotype-matched immunoglobulin G as a negative control, and blue histogram indicate cell stained with each antibodies.

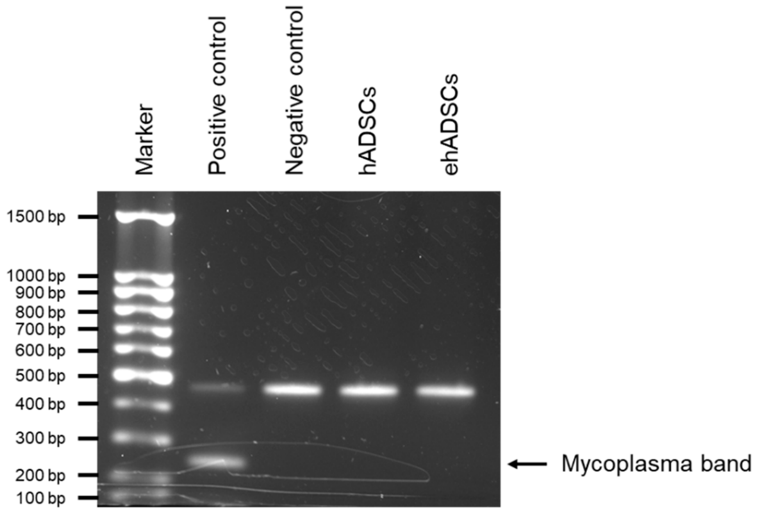

Supplemental Figure S2. Confirmation of mycoplasma contamination in hADSCs and ehADSCs.

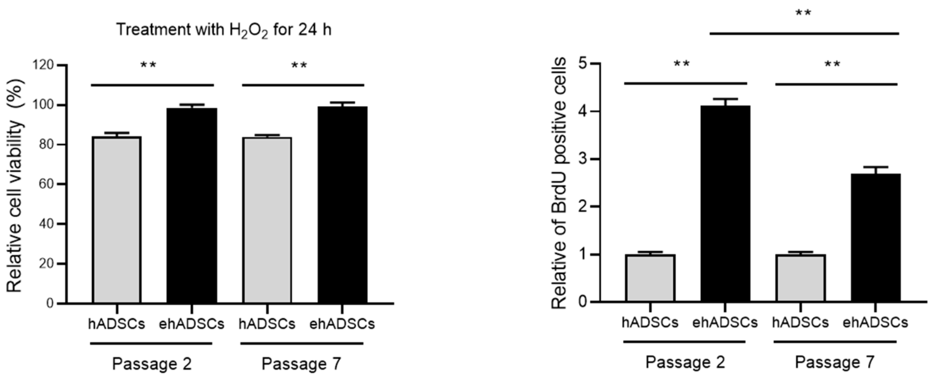

Supplemental Figure S3. The survival assay against oxidative stress and the proliferation assay between early passage (Passage 2) and late passage (Passage 7) in hADSCs and ehADSCs.
